# Wheat SWI3B Subunit of SWI/SNF Chromatin Remodeling Complex Governs Powdery Mildew Susceptibility by Suppressing Salicylic Acid Biosynthesis

**DOI:** 10.3390/jof12010068

**Published:** 2026-01-14

**Authors:** Wanzhen Chen, Yixian Fu, Mengdi Zhang, Wenrui Zhao, Pengfei Zhi, Cheng Chang

**Affiliations:** College of Life Sciences, Qingdao University, Qingdao 266071, China

**Keywords:** wheat, powdery mildew resistance, epigenetic regulation, TaSWI3B, *TaSARD1*, *TaICS1*

## Abstract

The fungal pathogen *Blumeria graminis forma specialis tritici* (*B.g. tritici*) infects bread wheat (*Triticum aestivum* L.) to cause wheat powdery mildew disease. Elucidating the molecular mechanism underlying wheat susceptibility to the pathogenic fungus *B.g. tritici* could facilitate wheat genetic improvement. In this study, we identified the wheat *TaSWI3B* gene as a novel *Susceptibility* gene positively regulating wheat susceptibility to *B.g. tritici.* The *TaSWI3B* gene encodes the SWI3B subunit of the SWI/SNF chromatin remodeling complex. The overexpression of the *TaSWI3B* gene enhances wheat powdery mildew susceptibility, whereas *TaSWI3B* silencing results in attenuated wheat powdery mildew susceptibility. Importantly, we found that TaSWI3B could be enriched at the promoter regions of the salicylic acid (SA) biosynthesis activator gene *TaSARD1*, facilitating nucleosome occupancy and thereby suppressing *TaSARD1* transcription and inhibiting SA biosynthesis. Silencing of *TaSARD1* and *TaICS1* encoding a key enzyme in SA biosynthesis could attenuate the SA biosynthesis and powdery mildew resistance potentiated by knockdown of *TaSWI3B* expression. Collectively, these results suggest that the SWI3B subunit of the wheat SWI/SNF chromatin remodeling complex negatively regulates SA biosynthesis by suppressing *TaSARD1* transcription at the epigenetic level and thus facilitates wheat powdery mildew susceptibility.

## 1. Introduction

As one of the most important wheat diseases, powdery mildew disease negatively affects both grain yield and quality [1,2,3]. The causal agent of wheat powdery mildew disease, *Blumeria graminis forma specialis tritici* (*B.g. tritici*), is the obligate biotrophic fungal pathogen [1,2,3]. *B.g. tritici* infection mainly relies on its air-borne conidia and could occur on any of the wheat aerial organs like the leaf blade, leaf sheath, stem, and even spike [1,2,3]. Upon landing on the wheat epidermal surface, *B.g. tritici* conidia germinate to form the germ tube, then penetrate the plant cell wall to generate the feeding structure haustorium, and finally develop into a microcolony to disperse more conidia for further infection [1,2,3]. Elucidating the molecular mechanism underlying the compatible wheat–*B.g. tritici* interaction could facilitate wheat genetic improvement for powdery mildew resistance [1,2,3].

In Eukaryotes, approximately 147 base pairs of DNA are wrapped around a histone octamer and packaged into the nucleosome [4]. The chromatin remodeling complex could utilize the ATP hydrolysis energy to move or alter the histone octomer, thereby changing the nucleosomal composition and occupancy to affect gene transcription [5,6]. As a highly conserved chromatin remodeling complex, SWI/SNF (SWItch/Sucrose Non-Fermentable) is composed of SNF2-type ATPases BRAHMA (BRM) or SPLAYED (SYD), as well as other core subunits like SWI3B and SWI3C [7,8]. The Arabidopsis AtSWI3B subunit of the SWI/SNF chromatin remodeling complex was reported to regulate various processes in plant development and environmental adaptation [9,10]. For instance, AtSWI3B associates with the protein phosphatase type 2C HYPERSENSITIVE TO ABA1 (HAB1) to regulate Abscisic acid (ABA) signaling [9]. In addition, AtSWI3B could interact with the histone deacetylase HDA6 to maintain transposon silencing [10]. However, whether and how SWI3B regulates plant–microbe interaction, especially the compatible wheat–*B.g. tritici* interaction, remains to be disclosed.

In this study, we characterized the function of wheat SWI3B in the compatible wheat–*B.g. tritici* interaction. Our results revealed that TaSWI3B maintains repressive chromatin state at the SA biosynthesis activator gene *TaSARD1* and negatively regulates wheat SA biosynthesis, thereby positively regulating the wheat powdery mildew susceptibility. Genetically manipulating the novel *susceptibility* gene *TaSWI3B* could contribute to wheat breeding against powdery mildew disease.

## 2. Materials and Methods

### 2.1. Plant and Fungal Materials

The *B.g. tritici*-susceptible wheat cultivar Yannong 999 and the virulent *B.g. tritici* genotype isolate E09 were employed for the analysis of the compatible wheat–*B.g. tritici* interaction in this study. Wheat seedlings were grown in climate chambers under a 16 h light/8 h dark with 20 °C/18 °C day/night cycle. The *B.g. tritici* isolate E09 was maintained on the leaves of Yannong 999 seedlings under a 20 °C day/18 °C night cycle.

### 2.2. Reverse Transcription Quantitative Polymerase Chain Reaction (RT-qPCR) and Nuclear Run-On Assays

The newly grown wheat leaves (n = 5, randomly chosen) with virus symptoms about two weeks post *BSMV* infection were harvested for the RT-qPCR and nuclear run-on assays. For the RT-qPCR assay, the total RNA was extracted from the wheat leaves using TRizol solution and treated with RNase-free DNase I to remove potential DNA contamination. The first-strand cDNA was synthesized using 1 μg of the total RNA and used as a template to detect the expression of the indicated wheat gene in the in the RT-qPCR. The RT-qPCR assay was performed using the ABI step-one real-time PCR system with the GoTaq qPCR Master Mix. The expression of *TaEF1* was set as the internal control, and the expression levels of *TaSWI3B*, *TaSARD1*, *TaICS1*, *TaPR1*, or *TaPR2* were measured by qPCR using the qPCR Master Mix (Invitrogen, Carlsbad, CA, USA) under the following programs: 95 °C for 3 min, 40 cycles at 95 °C for 20 s, 56 °C for 30 s, and 72 °C for 15 s, followed by 72 °C for 1 min. For the nuclear run-on assay, wheat cell nuclei were isolated and mixed with reaction buffer (25 mM biotin-16-UTP and 0.75 mM of ATP, CTP, and GTP) for the transcription reaction. After RNA extraction, the nascent RNA was enriched by streptavidin magnetic beads and subjected to the RT-qPCR assay. In the RT-qPCR and nuclear run-on assays, *TaSWI3B* is analyzed using the primers 5′TCCACTGCCGGTACCCTCAAGA3′/5′AACCTCTTGACGAGGGTGTTG3′.

### 2.3. BSMV-Mediated Gene Silencing

For the *BSMV*-VIGS assay, fragments of *TaSWI3B* were amplified using the primers 5′AAGGAAGTTTAATTAACTCATGGTGATAATAGG3′/5′AACCACCACCACCGTACGATTTACTCAAAGAGGCATC3′ and cloned into the pCa-γbLIC vector through the ligation-independent cloning technique to create construct *BSMV*-*TaSWI3B* as described by Yuan et al. [11]. The *BSMV*-*TaSARD1* and *BSMV*-*TaICS1* constructs were derived from previous studies [12]. The *BSMV*-VIGS assay was conducted as previously described [11], and leaves of BSMV-γ (BSMV-VIGS empty vector)-infected wheat plants were included as the negative control.

### 2.4. Single-Cell Transient Gene Overexpression Assay

For the single-cell transient gene overexpression assay, coding regions of *TaSWI3B-6A*, *TaSWI3B-6B*, and *TaSWI3B-6D* were amplified using primers 5′GGGGACAAGTTTGTACAAAAAAGCAGGCTTC ATGGCCACACCGCCGGCTCC3′/5′GGGGACCACTTTGTACAAGAAAGCTGGGTCTTAACTCATGGTGATAATAG3′, 5′GGGGACAAGTTTGTACAAAAAAGCAGGCTTCATGGCCACACCGCCGGCTCC3′/5′GGGGACCACTTTGTACAAGAAAGCTGGGTCTTAACTCATGGTGATAATAG3′, 5′GGGGACAAGTTTGTACAAAAAAGCAGGCTTCATGGCCACACCGCCGGCTCC3′/5′GGGGACCACTTTGTACAAGAAAGCTGGGTCTTAACTCATGGTGATAATAG3′, and PCR products were cloned into the pIPKb001 to create the pIPKb001-*TaSWI3B-6A* (for OE-*TaSWI3B-6A*), pIPKb001-*TaSWI3B-6B* (for OE-*TaSWI3B-6B*), and pIPKb001-*TaSWI3B-6D* (for OE-*TaSWI3B-6D*) constructs using the GATEWAY cloning technology (Invitrogen). The single-cell transient gene overexpression assay was performed as previously described [12,13,14].

### 2.5. B.g. tritici Microcolony Index Analysis

For the *B.g. tritici* microcolony formation analysis, newly grown upper leaves with virus symptoms were collected and subjected to inoculation with the *B.g. tritici* strain E09 conidia. About 72 h post *B.g. tritici* inoculation, leaf samples were fixed in ethanol–acetic acid solution (1:1, *v*/*v*) and kept in the destaining solution (lactic acid–glycerol–water, 1:1:1, *v*/*v*/*v*). Thereafter, *B.g. tritici*-infected leaves were stained with 0.1% (*w*/*v*) Coomassie brilliant blue R250 to visualize the fungal epiphytic structure under microscopy. About 1000 *B.g. tritici*–wheat interaction sites (randomly chosen) were analyzed in one experiment. The *B.g. tritici* conidial microcolony index was described as the percentage of the germinated *B.g. tritici* conidia with a microcolony.

### 2.6. B.g. tritici Haustorium Index Analysis

For the *B.g. tritici* haustorium formation analysis, the inoculation of *B.g. tritici* conidia spores was performed at least 16 h post bombardment. The leaf segments were stained for GUS activity 48 h post *B.g. tritici* spore inoculation and kept in the destaining solution. Before mounting for microscopy, the *B.g. tritici*-infected wheat leaves were stained with Coomassie blue to visualize the fungal epiphytic structure. About 50 *B.g. tritici*-infected wheat epidermal cells (randomly chosen) were analyzed in one experiment. The haustorium index was expressed as a percentage of GUS-staining cells with haustoria in the total GUS-staining cells attacked by germinated *B.g. tritici* conidia.

### 2.7. SA Measurement

Newly grown wheat leaves (n = 5) with virus symptoms about two weeks post *BSMV* infection were randomly collected and ground with liquid nitrogen into powder and then homogenized in 70% ethanol (*v*/*v*) containing the internal standard ortho-anisic acid. After centrifugation, the supernatant was collected and the pellet was homogenized with 90% *v*/*v* methanol. After centrifugation, both supernatants were pooled and evaporated under vacuum; 5% trichloroacetic acid was added to the remaining aqueous solution. After centrifugation, the supernatant was collected and mixed with ethyl acetate/cyclohexane. After centrifugation, the upper organic phase was collected. For SA quantification, organic phases were resuspended in HPLC starting solvent (methanol 40%, water 60%, acetic acid 1%) and analyzed by a reverse-phase HPLC column. The free SA amount was calculated in ng mg^−1^ fresh weight (FW) with reference to the amount of internal standard.

### 2.8. ChIP Assay and Nucleosomal Occupancy Analysis

The ChIP assay analyzing the enrichment of the TaSWI3B-HA protein at the *TaSARD1* gene promoter regions was conducted as previously described [12,13,14,15]. Briefly, α-HA antibodies (Santa Cruz Biotechnology, Dallas, TX, USA, sc-805) were employed for immunoprecipitation. DNA recovery after chromatin immunoprecipitation was quantified as the percentage of input. A nucleosome occupancy micrococcal nuclease (MNase) assay, analyzing chromatin assembly structure at *TaSARD1* promoter regions, was conducted as previously described. Briefly, wheat leaves were first cross-linked and then subjected to nuclear isolation and MNase digestion. Genomic DNA was then recovered and underwent qPCR analysis. Nuclei without MNase digestion treatment were employed as the input control. Primer sequences for qPCR analyzing the *TaSARD1* promoter were derived from previous studies.

### 2.9. Statistical Analysis

For the statistical analysis of gene transcription rates, transcript accumulation, SA measurement, nucleosomal occupancy at gene promoter regions, *B.g. tritici* microcolony formation, and *B.g. tritici* haustorium formation, three technical replicates per assay were analyzed using Student’s *t*-test, and the value represents the mean ± standard deviation. These assays were repeated in three independent biological replicates using dependently prepared samples with similar results.

## 3. Results

### 3.1. Homology-Based Identification of Wheat TaSWI3B Genes

**Figure 1:**
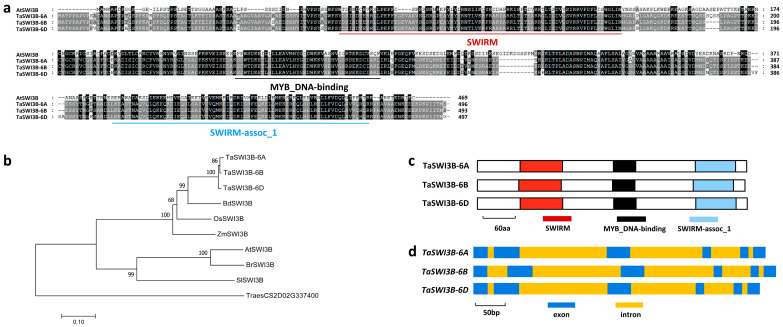
Identification of wheat TaSWI3B based on homology with Arabidopsis AtSWI3B. (**a**) Protein sequence alignments of Arabidopsis AtSWI3B, wheat TaSWI3B-6A, TaSWI3B-6B, and TaSWI3B-6D. (**b**) Phylogenetic relationships of the SWI3B proteins from Arabidopsis (At), mustard (Br), tomato (Sl), *Brachypodium* (Bd), maize (Zm), rice (Os), and wheat (Ta). (**c**) Domain arrangement of wheat TaSWI3B-6A, TaSWI3B-6B, and TaSWI3B-6D proteins. (**d**) Genomic sequence structure of wheat *TaSWI3B-6A*, *TaSWI3B-6B*, and *TaSWI3B-6D* genes.

In this study, we aimed to explore the putative regulation of the SWI3B subunit of the wheat SWI/SNF chromatin remodeling complex on the compatible wheat–*B.g. tritici* interaction. To this end, we first employed the amino acid sequence of Arabidopsis AtSWI3B (At2g33610) as a query to search the genome of hexaploid bread (*Triticum aestivum* L., AABBDD). *TaSWI3B-6A* (*TraesCS6A02G167800*), *TaSWI3B-6B* (*TraesCS6B02G195400*), and *TaSWI3B-6D* (*TraesCS6D02G156700*), separately located on wheat chromosomes 6A, 6B, and 6D, were identified as wheat homologs of AtSWI3B. These predicted TaSWI3B-6A, TaSWI3B-6B, and TaSWI3B-6D proteins shared more than 39% identity with Arabidopsis AtSWI3B (Figure 1a). As shown in the Supplementary Figure S1, SWI3B proteins from bread wheat, *Triticum urartu*, *Aegilops tauschii*, *Brachypodium distachyon*, barley, maize, and rice shared high similarity. Phylogenetic analysis further validated that wheat TaSWI3B-6A, TaSWI3B-6B, and TaSWI3B-6D proteins are wheat close homologs of Arabidopsis AtSWI3B, mustard BrSWI3B, tomato SlSWI3B, *Brachypodium* BdSWI3B, maize ZmSWI3B, and rice OsSWI3B (Figure 1b). As shown in Figure 1c, SWIRM, MYB_DNA-binding, and SWIRM-assoc_1 domains were identified from the N-terminal, middle, and C-terminal parts of all TaSWI3B proteins, respectively. The coding regions of *TaSWI3B-6A*, *TaSWI3B-6B*, and *TaSWI3B-6D* genomic sequences all contained six exons and five introns (Figure 1d).

### 3.2. Functional Characterization of TaSWI3B Gene in the Regulation of Compatible Wheat–B.g. tritici Interaction

**Figure 2:**
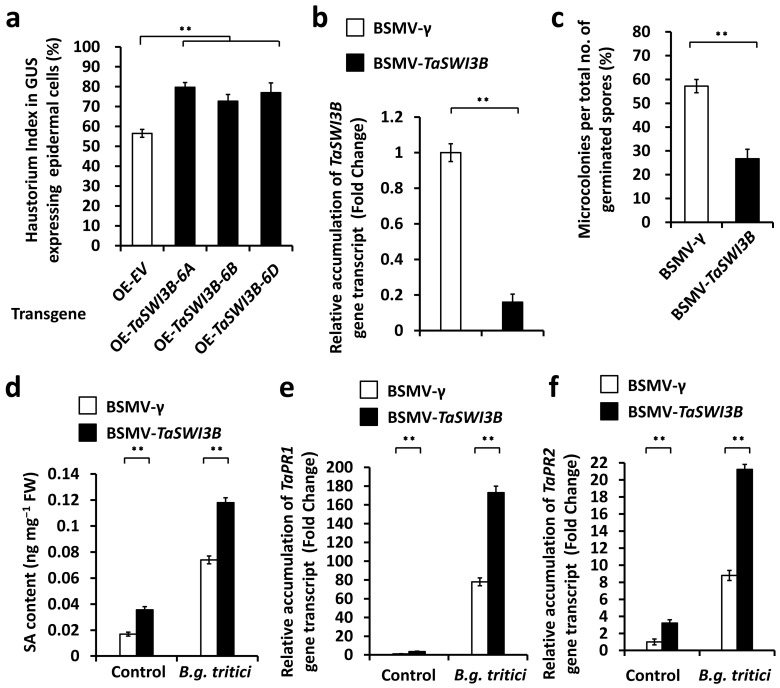
Functional characterization of the *TaSWI3B* gene in the regulation of compatible wheat–*B.g. tritici* interaction. (**a**) Analysis of *B.g. tritici* haustorium index in wheat epidermal cells overexpressing *TaSWI3B*. (**b**) RT-qPCR analysis of *TaSWI3B* gene expression levels in the wheat leaves silencing *TaSWI3B*. (**c**) Analysis of *B.g. tritici* microcolony index on wheat leaves silencing *TaSWI3B*. (**d**) Measurement of SA accumulation in the wheat leaves silencing *TaSWI3B*. RT-qPCR analysis of *TaPR1* (**e**) and *TaPR2* (**f**) gene expression levels in the wheat leaves silencing *TaSWI3B*. For (**b**–**f**), the leaves of BSMV-γ (BSMV-VIGS empty vector)-infected wheat plants were included as the negative control. For (**a**–**f**), three technical replicates per treatment were statistically analyzed, and data are presented as the mean ± SE (Student’s *t*-test; ** *p* < 0.01), and these assays were repeated in three independent biological replicates with similar results.

To characterize the function of *TaSWI3B* genes in the regulation of the compatible wheat–*B.g. tritici* interaction, we first employed transient gene expression assays to overexpress these *TaSWI3B-6A*, *TaSWI3B-6B*, and *TaSWI3B-6D* genes in the wheat leaf epidermal cells. Then, we inoculated on these bombarded wheat leaves with *B.g. tritici* conidia and statistically analyzed the formation of *B.g. tritici* haustoria. As shown in Figure 2a, the *B.g. tritici* haustorium index (HI%) increased from about 56.5% for the empty vector (OE-EV) control to above 72.6% on wheat cells overexpressing the *TaSWI3B-6A*, *TaSWI3B-6B*, or *TaSWI3B-6D* gene. Thereafter, we employed *barley stripe mosaic virus* (*BSMV*)-induced gene silencing (*BSMV*-VIGS) to silence all endogenous *TaSWI3B* genes in the wheat leaves. A fragment proximal to the 3′ end shared by the allelic *TaSWI3B-6A*, *TaSWI3B-6B*, and *TaSWI3B-6D* genes was chosen for the *BSMV*-VIGS, and another fragment proximal to the 5′ end conserved among the allelic *TaSWI3B-6A*, *TaSWI3B-6B*, and *TaSWI3B-6D* genes was chosen for the reverse transcription quantitative polymerase chain reaction (RT-qPCR) analysis (Supplementary Figure S2). As shown in Supplementary Figure S3, accumulation levels of the *TaHDA9* gene in wheat leaves significantly increased upon infection with the virulent *B.g. tritici* pathogen. A reverse transcription quantitative polymerase chain reaction (RT-qPCR) assay was performed to confirm efficient silencing of the *TaSWI3B*. As shown in Figure 2b, the accumulation level of *TaSWI3B* gene transcripts decreased significantly in wheat leaves silencing the *TaSWI3B* gene. To ensure the silencing specificity of *TaSWI3B* genes by *BSMV*-VIGS, we searched the whole genome of allohexaploid bread wheat using the fragments chosen for *TaSWI3B* silencing by the *BSMV*-VIGS method. As shown in Supplementary Figure S4, there is no putative off-target gene of *BSMV*-*TaSWI3B*. *B.g. tritici* conidia were inoculated on these *BSMV*-VIGS wheat leaves, and the formation of *B.g. tritici* microcolony was statistically analyzed. As shown in Figure 2c, the *B.g. tritici* microcolony index (MI%) decreased from 58.2% for the control plants (*BSMV*-γ) to 37.7% for *TaSWI3B*-silenced (*BSMV*-*TaSWI3Bas*) plants. These HI% and MI% data suggest that the *TaSWI3B* gene negatively regulates wheat powdery mildew resistance and positively contributes to *B.g. tritici* post-penetration events like haustorial development and microcolony formation.

It is well known that phytohormone salicylic acid (SA) plays important roles in wheat post-penetration resistance against powdery mildew disease [12,13,14,15,16,17]. To analyze the potential regulation of *TaSWI3B* genes on SA accumulation, we first conducted a *BSMV*-VIGS assay to silence all endogenous *TaSWI3B* genes in the wheat leaves, inoculated these *BSMV*-VIGS wheat leaves with *B.g. tritici* conidia, and performed High-Performance Liquid Chromatography (HPLC) to analyze the SA accumulation. As shown in Figure 2d, the SA accumulation level significantly increased in the *TaSWI3B*-silenced wheat leaves, compared with that of *BSMV*-γ control plants, indicating that TaSWI3B negatively regulates the SA accumulation in bread wheat. Consistent with this, accumulation levels of SA signaling marker gene *TaPR1* and *TaPR2* transcripts were remarkably enhanced by silencing of *TaSWI3B* (Figure 2e,f). These results suggested that the TaSWI3B subunit of the wheat SWI/SNF chromatin remodeling complex negatively regulates SA biosynthesis and contributes to the wheat susceptibility to the fungal pathogen *B.g. tritici*.

### 3.3. Epigenetic and Transcriptional Regulation of SA Biosynthesis Activator Gene TaSARD1 by TaSWI3B

**Figure 3:**
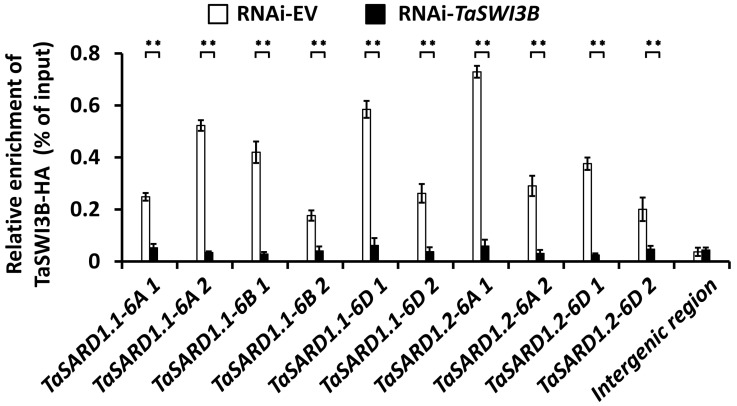
Analysis of TaSWI3B enrichment at *TaSARD1* promoters. ChIP-qPCR analysis of TaSWI3B-HA enrichment at *TaSARD1* promoter regions in the wheat cells. Three technical replicates per treatment were statistically analyzed, and data are presented as the mean ± SE (Student’s *t*-test; ** *p* < 0.01), and these assays were repeated in three independent biological replicates with similar results.

It was recently reported that the SA biosynthesis activator gene *TaSARD1* plays a key role in wheat powdery mildew resistance, and *TaSARD1* gene transcription is tightly regulated at chromatin levels [12,13,14,15]. We ask whether the TaSWI3B subunit of the wheat SWI/SNF chromatin remodeling complex could be enriched at the *TaSARD1* promoters. To this end, we first transfected the wheat protoplast with *TaSWI3B-HA* constructs and performed a ChIP assay to characterize the distribution of TaSWI3B-HA at *TaSARD1* promoters (Figure 3). *TaSARD1* promoter regions, previously demonstrated to be regulated by epigenetic events like histone acetylation and chromatin assembly, were chosen for the ChIP analysis. As shown in Figure 3, these *TaSARD1* promoter regions were found to be enriched in DNA samples immunoprecipitated with the antibody specifically against TaSWI3B-HA, directly indicating that the TaSWI3B subunit of the wheat SWI/SNF chromatin remodeling complex is enriched at promoter regions of the *TaSARD1* genes.

**Figure 4:**
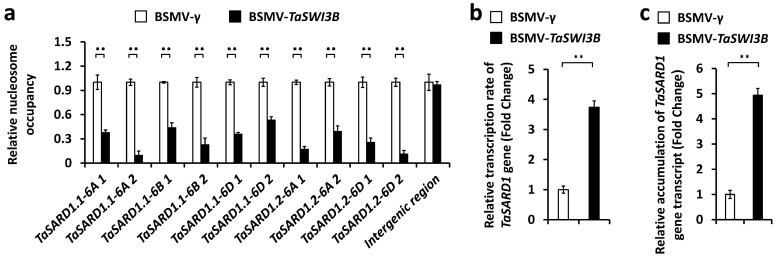
Characterization of nucleosomal occupancy and gene transcription at *TaSARD1* loci in *TaSWI3B*-silenced wheat leaves. (**a**) MNase analysis of nucleosome occupancy at *TaSARD1* promoters in the wheat leaves silencing *TaSWI3B*. The nucleosome occupancy levels in wheat leaves infected with the *BSMV*-γ empty vector (negative control) were set to 1.0. Transcription rates (**b**) and expression levels (**c**) of the *TaSARD1* gene in the wheat leaves silencing *TaSWI3B* were measured by nuclear run-on and qRT-PCR assays, respectively. Leaves of BSMV-γ (BSMV-VIGS empty vector) infected wheat plants were included as the negative control. For a, b, and c, three technical replicates per treatment were statistically analyzed, and data are presented as the mean ± SE (Student’s *t*-test; ** *p* < 0.01), and these assays were repeated in three independent biological replicates with similar results.

To examine whether the TaSWI3B subunit of the wheat SWI/SNF chromatin remodeling complex affects chromatin structure at *TaSARD1* promoter regions, we performed the micrococcal nuclease (MNase) assay. By enzymatic cleavage of linker DNA connecting nucleosomes, MNase assays could implicate nucleosome occupancy. As shown in Figure 4a, silencing of the *TaSWI3B* gene resulted in significantly reduced nucleosome occupancy at *TaSARD1* promoters. Nuclear run-on and qRT-PCR assays demonstrated that silencing of the *TaSWI3B* gene led to a significantly increased transcription rate and transcript accumulation of the *TaSARD1* gene (Figure 4b,c). These results suggested that the TaSWI3B subunit of the wheat SWI/SNF chromatin remodeling complex suppresses *TaSARD1* gene transcription, probably via maintaining a repressive chromatin state at the *TaSARD1* gene.

### 3.4. Functional Analysis of TaSARD1-TaICS1-SA Circuit in the Regulation of TaSWI3B-Governed Wheat–B.g. tritici Interaction

**Figure 5:**
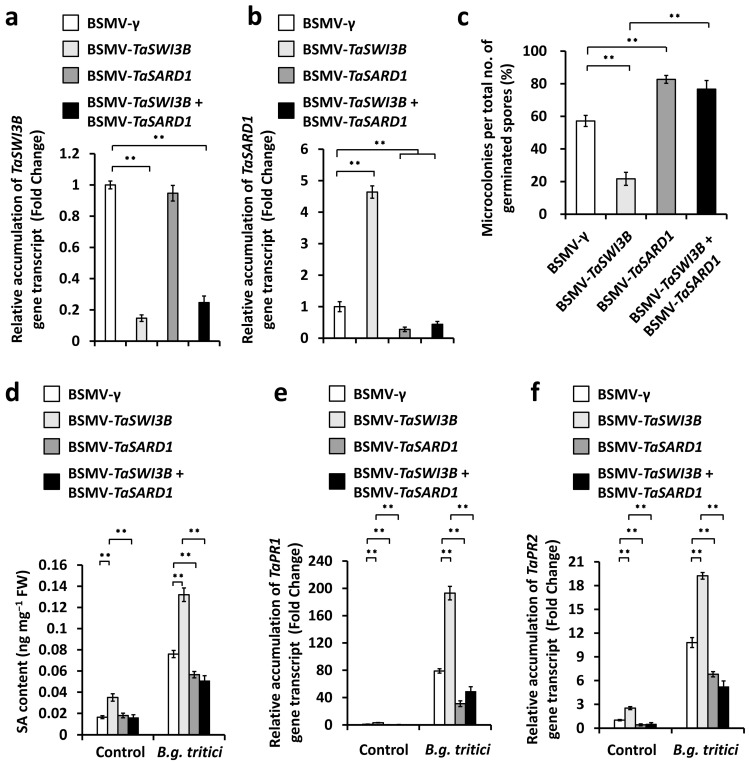
Characterization of the genetic interplay of *TaSWI3B* and *TaSARD1* in the regulation of compatible wheat–*B.g. tritici* interaction. RT-qPCR analysis of *TaSWI3B* (**a**) and *TaSARD1* (**b**) expression levels in the wheat leaves silencing *TaSWI3B*, *TaSARD1*, or co-silencing *TaSWI3B* and *TaSARD1*. (**c**) Analysis of *B.g. tritici* microcolony index on wheat leaves silencing *TaSWI3B*, *TaSARD1*, or co-silencing *TaSWI3B* and *TaSARD1*. (**d**) Measurement of SA accumulation in the wheat leaves silencing *TaSWI3B*, *TaSARD1*, or co-silencing *TaSWI3B* and *TaSARD1*. RT-qPCR analysis of *TaPR1* (**e**) and *TaPR2* (**f**) gene expression levels in the wheat leaves silencing *TaSWI3B*, *TaSARD1*, or co-silencing *TaSWI3B* and *TaSARD1*. For (**a**–**f**), leaves of BSMV-γ (BSMV-VIGS empty vector)-infected wheat plants were included as the negative control, and three technical replicates per treatment were statistically analyzed, and data are presented as the mean ± SE (Student’s *t*-test; ** *p* < 0.01). These assays were repeated in three independent biological replicates with similar results.

We ask whether the characterized *TaSARD1*-activated SA biosynthesis contributes to wheat powdery mildew resistance suppressed by the TaSWI3B subunit of the wheat SWI/SNF chromatin remodeling complex. To examine this hypothesis, we conducted the *BSMV*-VIGS assay to simultaneously silence *TaSWI3B* and *TaSARD1* genes in the wheat leaves, inoculated these *BSMV*-VIGS wheat leaves with *B.g. tritici* conidia, and statistically analyzed the formation of a *B.g. tritici* microcolony. As shown in Figure 5a,b, accumulation levels of *TaSWI3B* or *TaSARD1* gene transcript decreased remarkably in wheat leaves co-silencing *TaSWI3B* and *TaSARD1* genes, compared with the *BSMV-γ* control. The *B.g. tritici* MI% increased to above 77.7% in wheat leaves co-silencing *TaSWI3B* and *TaSARD1* genes, compared with 57.5% for the *BSMV*-γ control plants (Figure 5c). The SA accumulation level significantly decreased in wheat leaves co-silencing *TaSWI3B* and *TaSARD1* genes, compared with the *BSMV*-γ control plants (Figure 5d). Consistent with this, simultaneous silencing of *TaSWI3B* and *TaSARD1* genes resulted in a significant reduction in accumulation levels of *TaPR1* and *TaPR2* transcripts, compared with the *BSMV-γ* control (Figure 5e,f). These data collectively suggested that the TaSWI3B subunit of the wheat SWI/SNF chromatin remodeling complex negatively regulated SA biosynthesis, probably via epigenetic suppression of the *TaSARD1* gene, thereby contributing to wheat susceptibility to the *B.g. tritici* pathogen.

**Figure 6:**
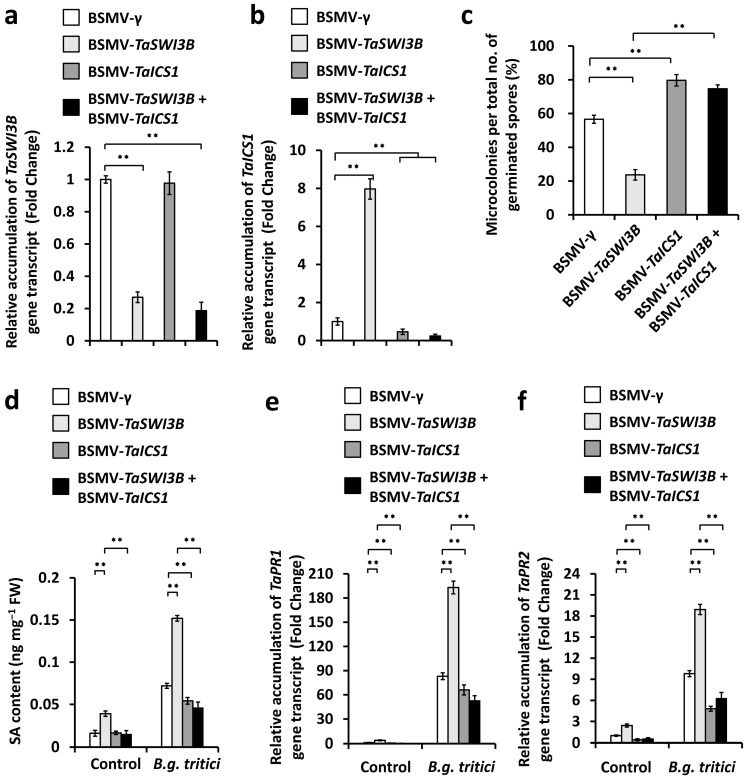
Characterization of the genetic interplay of *TaSWI3B* and *TaICS1* in the regulation of compatible wheat–*B.g. tritici* interaction. RT-PCR analysis of *TaSWI3B* (**a**) and *TaICS1* (**b**) expression levels in the wheat leaves silencing *TaSWI3B*, *TaICS1*, or co-silencing *TaSWI3B* and *TaICS1*. (**c**) Analysis of *B.g. tritici* microcolony index on wheat leaves silencing *TaSWI3B*, *TaICS1*, or co-silencing *TaSWI3B* and *TaICS1*. (**d**) Measurement of SA accumulation in the wheat leaves silencing *TaSWI3B*, *TaICS1*, or co-silencing *TaSWI3B* and *TaICS1*. RT-qPCR analysis of *TaPR1* (**e**) and *TaPR2* (**f**) gene expression levels in the wheat leaves wheat leaves silencing *TaSWI3B*, *TaICS1*, or co-silencing *TaSWI3B* and *TaICS1*. For (**a**–**f**), leaves of BSMV-γ (BSMV-VIGS empty vector)-infected wheat plants were included as the negative control, and three technical replicates per treatment were statistically analyzed, and data are presented as the mean ± SE (Student’s *t*-test; ** *p* < 0.01). These assays were repeated in three independent biological replicates with similar results.

TaICS1 (isochorismate synthase 1) was identified as a core component of wheat SA biosynthetic machinery [12,13,14,15]. We ask whether the characterized *TaICS1*-mediated SA biosynthesis contributes to wheat powdery mildew resistance suppressed by the TaSWI3B subunit of the wheat SWI/SNF chromatin remodeling complex. To examine this hypothesis, we conducted a *BSMV*-VIGS assay to simultaneously silence *TaSWI3B* and *TaICS1* genes in the wheat leaves, inoculated these *BSMV*-VIGS wheat leaves with *B.g. tritici* conidia, and statistically analyzed the formation of a *B.g. tritici* microcolony. As shown in Figure 6a,b, accumulation levels of *TaSWI3B* or *TaICS1* gene transcripts decreased remarkably in wheat leaves co-silencing *TaSWI3B* and *TaICS1* genes, compared with the *BSMV-γ* control. The *B.g. tritici* MI% increased to above 74.8% in wheat leaves co-silencing the *TaSWI3B* and *TaICS1* genes, compared with 56.6% for the *BSMV*-γ control plants (Figure 6c). The SA accumulation level significantly decreased in wheat leaves with the co-silencing of *TaSWI3B* and *TaICS1* genes, compared with the *BSMV*-γ control plants (Figure 6d). Consistent with this, simultaneous silencing of *TaSWI3B* and *TaICS1* genes resulted in a significant reduction in accumulation levels of *TaPR1* and *TaPR2* transcripts, compared with the *BSMV-γ* control (Figure 6e,f). These results collectively implicated that the TaSWI3B subunit of the wheat SWI/SNF chromatin remodeling complex negatively regulates SA biosynthesis mediated by the TaSARD1-TaICS1 module and thus facilitates powdery mildew susceptibility.

## 4. Discussion

### 4.1. S Gene TaSWI3B Facilitates Wheat Powdery Mildew Susceptibility

In this study, *TaSWI3B-6A*, *TaSWI3B-6B*, and *TaSWI3B-6D*, separately located on wheat chromosomes 6A, 6B, and 6D, were identified as *AtSWI3B* homologs in hexaploidy bread (*Triticum aestivum* L., AABBDD). Transient overexpression of *TaSWI3B-6A*, *TaSWI3B-6B*, and *TaSWI3B-6D* in wheat epidermal cells resulted in increased *B.g. tritici* HI%, whereas silencing of *TaSWI3B* caused decreased *B.g. tritici* MI%, indicating that the TaSWI3B subunit of the wheat SWI/SNF chromatin remodeling complex negatively regulates powdery mildew resistance and facilitates haustorial development and microcolony formation of the *B.g. tritici* pathogen. Multiple epigenetic regulators have been demonstrated to finetune the compatible wheat–*B.g. tritici* interaction. For instance, wheat DNA methyltrasferase TaMET1 suppresses SA biosynthesis to negatively regulate wheat powdery mildew resistance, while wheat histone acetyltransferase TaHAG1 activates the SA signaling regulator gene *TaPAD4* and positively contributes to powdery mildew resistance [15,18]. Wheat chromatin assembly factor-1 (CAF-1) suppresses the *TaSARD1* and wax biosynthesis gene *TaECR* to control powdery mildew susceptibility [12]. Herein, we demonstrated that *TaSWI3B* expression is induced by *B.g. tritici* infection. In contrast, expression of TaSWP73 encoding another component of the wheat SWI/SNF chromatin remodeling complex did not respond to *B.g. tritici* infection [13].

### 4.2. TaSWI3B Negatively Regulates SA Biosynthesis

Plant hormone SA plays a key role in wheat powdery mildew resistance, and *TaSARD1* was previously identified as an activator of SA biosynthesis [12,13,14,15,16,17,18]. In this study, we demonstrated that the TaSWI3B subunit of the wheat SWI/SNF chromatin remodeling complex is enriched at promoter regions of the *TaSARD1* genes and suppresses *TaSARD1* gene transcription via maintaining a repressive chromatin state at the *TaSARD1* gene. Consistent with this, SA overaccumulation and defense-marker gene activation were observed in the *TaSWI3B*-silenced wheat plants. Interestingly, silencing of *TaSARD1* and the SA biosynthesis gene *TaICS1* could attenuate the SA biosynthesis and powdery mildew resistance potentiated by the knockdown of *TaSWI3B* expression. This study allows us to propose a model of how the SWI3B subunit of SWI/SNF chromatin remodeling complex positively regulates wheat susceptibility to the *B.g. tritici* pathogen. In this model, the wheat SWI3B subunit of the SWI/SNF chromatin remodeling complex is enriched at the *TaSARD1* gene to enhance nucleosomal occupancy and suppress *TaSARD1* gene transcription. Consequently, SA biosynthesis mediated by the TaSARD1-TaICS1 module is maintained at a low level, resulting in relatively high susceptibility to the *B.g. tritici* pathogen. In the absence of the wheat SWI3B subunit of the SWI/SNF chromatin remodeling complex, nucleosomal occupancy at the *TaSARD1* gene is decreased and *TaSARD1* gene transcription is activated. As a result, SA biosynthesis mediated by the *TaSARD1*-*TaICS1* module is stimulated, leading to compromised susceptibility to the *B.g. tritici* pathogen. Wheat chromatin assembly factor CAF-1 was previously identified as an epigenetic suppressor of SA biosynthesis [12]. Therefore, it is intriguing to examine the potential interplay between TaSWI3B and CAF-1 in the epigenetic regulation of SA biosynthesis.

### 4.3. Potential Exploitation of TaSWI3B in Wheat Resistance Breeding

Previous studies have identified a plethora of *susceptibility* (*S*) genes that facilitate wheat compatibility with adapted pathogens [19,20,21]. These *S* genes could regulate various processes in wheat–pathogen interaction, including pathogen penetration, plant defense, and even pathogen sustenance [19,20,21]. For instance, wheat S genes *TaFAS1, TaFAS2, TaMSI1*, *TaMET1*, *TaSWP73*, and *TaSWI3D* suppress wheat defense response against *B.g. tritici* [12,13,14,15]. In contrast, wheat S genes *TaAMT2;3a* and *TaSTP3/6/13*, respectively, encode ammonium and sugar transporter to facilitate the nutrient uptake and sustenance of the wheat stripe rust pathogen [22,23,24,25,26,27]. In this study, we demonstrated that the *S* gene *TaSWI3B* negatively regulates the biosynthesis of defense hormone SA, thereby contributing to the compatible wheat–*B.g. tritici* interaction.

As discussed by prior reviews, genetic manipulation of S genes by genome editing and TILLING techniques could confer to wheat durable and broad-spectrum disease resistance [28,29,30,31,32,33]. For instance, genome editing of S genes *TaWRKY19* and *TaPsIPK1* using CRISPR (clustered regularly interspaced short palindromic repeats)-Cas9 (CRISPR-associated 9) systems confers to wheat resistance against wheat stripe rust disease [34,35]. Similarly, editing of wheat S genes *TaMLO* and *TaEDR1* by CRISPR-Cas9 and transcription activator-like effector nucleases (TALENs) conferred wheat resistance against *B.g. tritici* [36,37]. Knockout of the *S* gene *TaSWI3B* by CRISPR-Cas9 and TILLING techniques represents a promising direction in wheat breeding against powdery mildew disease.

## 5. Conclusions

In this study, we explored the potential regulation of the SWI3B subunit of the SWI/SNF chromatin remodeling complex in the compatible wheat–*B.g. tritici* interaction. We revealed that the TaSWI3B subunit of the wheat SWI/SNF chromatin remodeling complex maintains epigenetic suppression of the SA biosynthesis activator gene *TaSARD1* and negatively regulates SA biosynthesis, thereby facilitating wheat susceptibility to the *B.g. tritici* pathogen. Therefore, the *TaSWI3B* gene was identified as a novel *S* gene suppressing wheat post-penetration resistance against powdery mildew. Characterizing the potential pleiotropic effects of *TaSWI3B* gene on other wheat agronomic traits might contribute to its exploitation in wheat powdery mildew resistance breeding. In addition, the TaWI3B subunit of the SWI/SNF chromatin remodeling complex was identified as a novel regulator of wheat SA biosynthesis and powdery mildew resistance. Characterizing potential regulation of the integral SWI/SNF chromatin remodeling complex on the compatible wheat–*B.g. tritici* interaction might provide more insights into the epigenetic regulation of SA biosynthesis and plant–fungal pathogen interaction in future study.

## Data Availability

The original contributions presented in this study are included in the article/Supplementary Materials. Further inquiries can be directed to the corresponding author.

## Supplementary Materials

The following supporting information can be downloaded at https://www.mdpi.com/article/10.3390/jof12010068/s1. Supplementary Figure S1. Protein sequence alignments of SWI3B from bread wheat (Ta), *Triticum urartu* (Tu), *Aegilops tauschii* (At), *Brachypodium distachyon* (Bd), barley (Hv), maize (Zm), and rice (Os). Supplementary Figure S2. Alignment of nucleotide sequences at the coding regions of allelic *TaSWI3B-6A*, *TaSWI3B-6B*, and *TaSWI3B-6D* genes. Supplementary Figure S3. RT-qPCR analysis of *TaSWI3B* expression levels in wheat leaves under *B.g. tritici* infection. Supplementary Figure S4. Whole-genome searching of allohexaploid bread wheat using the fragments chosen for *TaSWI3B* silencing by the *BSMV*-VIGS method in this study. Supplementary Figure S5. Powdery mildew microcolony formation on wheat plants infected with BSMV-γ and BSMV-TaSWI3B. Bar, 150 μm.

## Abbreviations

The following abbreviations are used in this manuscript:

SWI3	SWITCH subunit 3
SARD1	SAR Deficient 1
ICS1	Isochorismate synthase 1
*B.g. tritici*	*Blumeria graminis forma specialis tritici*
SA	Salicylic acid
SWI/SNF	SWItch/Sucrose Non-Fermentable
RT-qPCR	Reverse transcription quantitative polymerase chain reaction
ChIP	Chromatin immunoprecipitation
MNase	Micrococcal nuclease
