# Supplementary material for: Wheat SWI3B Subunit of SWI/SNF Chromatin Remodeling Complex Governs Powdery Mildew Susceptibility by Suppressing Salicylic Acid Biosynthesis
Source: J Fungi (Basel). 2026 Jan 14;12(1):68. doi: 10.3390/jof12010068 (PMC12842968; doi:10.3390/jof12010068)
Supplement: Supplementary file 1 [file jof-12-00068-s001.zip › jof-3989485-supplementary.pdf]

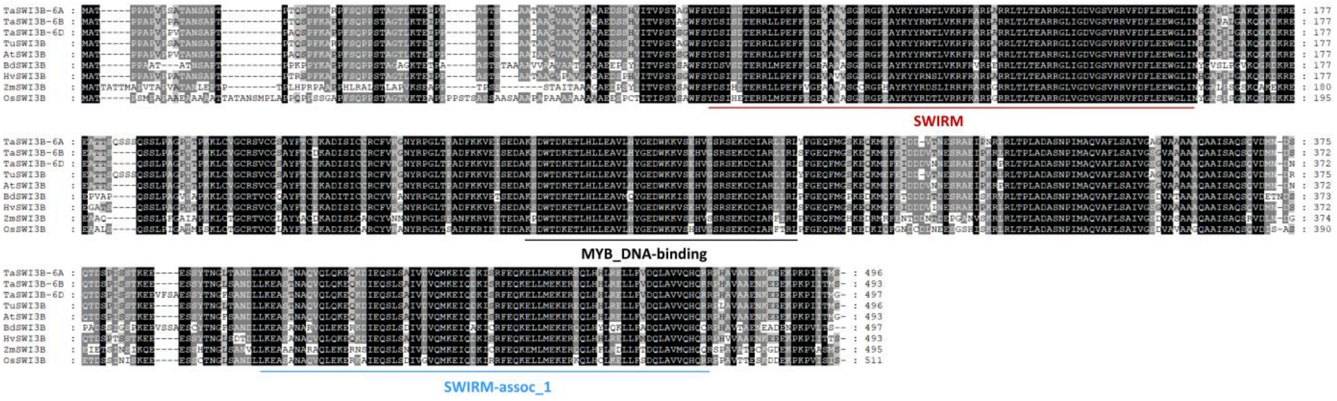

Supplementary Figure S1 Protein sequence alignments of SWI3B from bread wheat (Ta), *Triticum urartu* (Tu), *Aegilops tauschii* (At) *Brachypodium distachyon* (Bd), barley (Hv), maize (Zm), rice (Os).

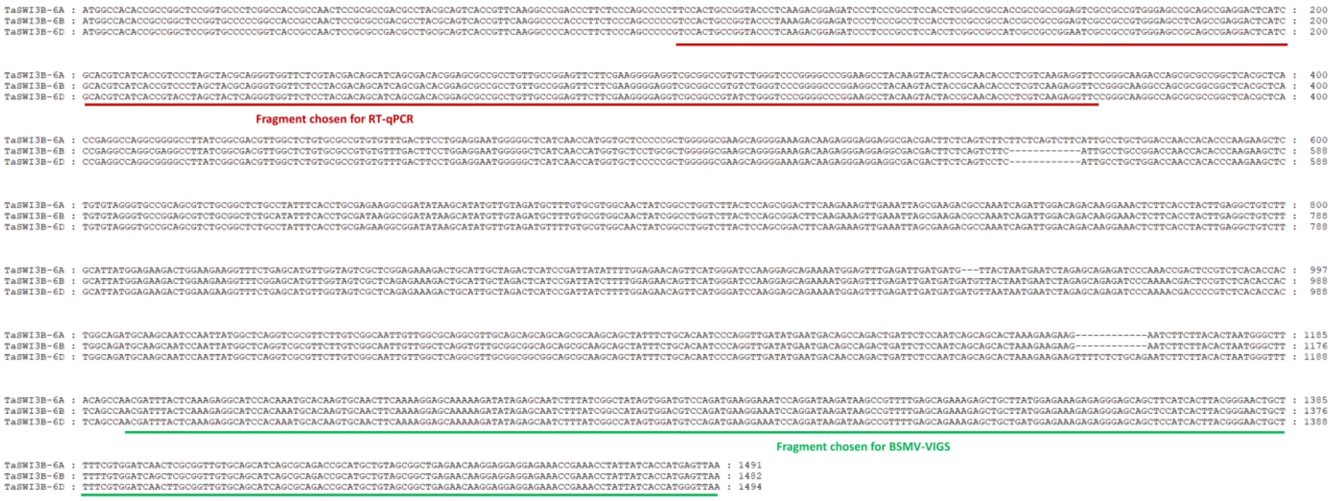

Supplementary Figure S2 Alignment of nucleotide sequences at the coding regions of allelic *TaSWI3B-6A*, *TaSWI3B-6B*, and *TaSWI3B-6D* genes**.** Regions chosen for the RT-qPCR and BSMV-VIGS are underlined in blue and green, respectively.

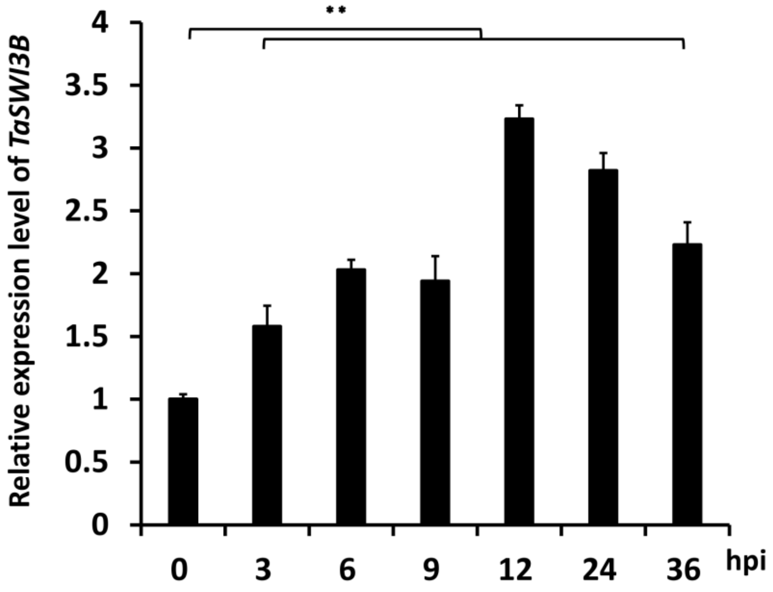

Supplementary Figure S3 RT-qPCR analysis of *TaSWI3B* expression levels in wheat leaves under *B.g. tritici* infection**.**

Results table ⊟

Show All entries | Show/hide columns (4 hidden) | Filter

Genomic Location	Overlapping Gene(s)	Orientation	Length	Score	E-val	%ID
6A:172111802-172112005 [Sequence]	TraesCS6A02G167800	Reverse	204 [Sequence]	204	5.3e-110	100.0 [Alignment]
6B:231348425-231348628 [Sequence]	TraesCS6B02G195400	Reverse	204 [Sequence]	192	7.6e-103	98.5 [Alignment]
6D:133206969-133207172 [Sequence]	TraesCS6D02G156700	Reverse	204 [Sequence]	188	1.9e-100	98.0 [Alignment]
6A:172112089-172112186 [Sequence]	TraesCS6A02G167800	Reverse	98 [Sequence]	98	9.5e-47	100.0 [Alignment]
6D:133207264-133207361 [Sequence]	TraesCS6D02G156700	Reverse	98 [Sequence]	94	2.3e-44	99.0 [Alignment]
6B:231348712-231348809 [Sequence]	TraesCS6B02G195400	Reverse	98 [Sequence]	90	5.6e-42	98.0 [Alignment]
2B:368715717-368715737 [Sequence]		Reverse	21 [Sequence]	21	0.84	100.0 [Alignment]
2B:60095524-60095544 [Sequence]		Forward	21 [Sequence]	21	0.84	100.0 [Alignment]
1A:90986139-90986159 [Sequence]		Forward	21 [Sequence]	21	0.84	100.0 [Alignment]
1A:410695991-410696011 [Sequence]		Forward	21 [Sequence]	21	0.84	100.0 [Alignment]
2D:16136680-16136700 [Sequence]		Forward	21 [Sequence]	21	0.84	100.0 [Alignment]
2D:405299625-405299645 [Sequence]		Forward	21 [Sequence]	21	0.84	100.0 [Alignment]
6D:389657974-389657994 [Sequence]		Forward	21 [Sequence]	21	0.84	100.0 [Alignment]
6B:671623232-671623252 [Sequence]		Forward	21 [Sequence]	21	0.84	100.0 [Alignment]
1B:538862808-538862828 [Sequence]		Forward	21 [Sequence]	21	0.84	100.0 [Alignment]
3A:634334046-634334070 [Sequence]		Reverse	25 [Sequence]	21	0.84	96.0 [Alignment]
3A:420892278-420892298 [Sequence]		Forward	21 [Sequence]	21	0.84	100.0 [Alignment]
7A:248300523-248300547 [Sequence]		Forward	25 [Sequence]	21	0.84	96.0 [Alignment]
3B:84930028-84930048 [Sequence]		Reverse	21 [Sequence]	21	0.84	100.0 [Alignment]
7B:331263940-331263960 [Sequence]		Forward	21 [Sequence]	21	0.84	100.0 [Alignment]
1A:591274096-591274119 [Sequence]		Reverse	24 [Sequence]	20	3.3	95.8 [Alignment]
6A:167559690-167559709 [Sequence]		Reverse	20 [Sequence]	20	3.3	100.0 [Alignment]
6A:159424330-159424349 [Sequence]		Forward	20 [Sequence]	20	3.3	100.0 [Alignment]
6A:159505835-159505854 [Sequence]		Forward	20 [Sequence]	20	3.3	100.0 [Alignment]
1D:353459036-353459055 [Sequence]		Reverse	20 [Sequence]	20	3.3	100.0 [Alignment]
6B:222883030-222883049 [Sequence]		Reverse	20 [Sequence]	20	3.3	100.0 [Alignment]
1B:611527996-611528015 [Sequence]		Forward	20 [Sequence]	20	3.3	100.0 [Alignment]
4A:725029943-725029962 [Sequence]		Forward	20 [Sequence]	20	3.3	100.0 [Alignment]
7A:374183218-374183237 [Sequence]		Forward	20 [Sequence]	20	3.3	100.0 [Alignment]
7A:675765859-675765878 [Sequence]		Forward	20 [Sequence]	20	3.3	100.0 [Alignment]

Supplementary Figure S4 Whole genome searching of allohexaploid bread wheat using the fragments chosen for *TaSWI3B* silencing by BSMV-VIGS method in this study.

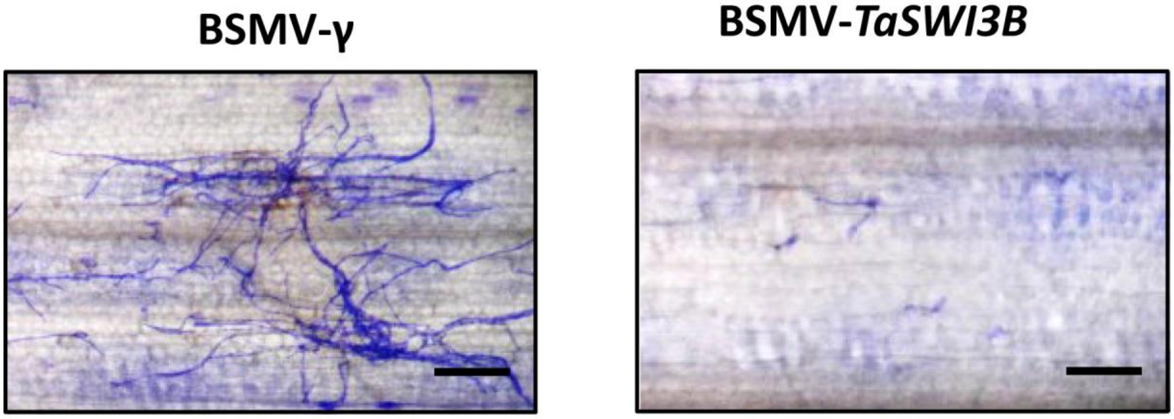

Supplementary Figure S5 Powdery mildew microcolony formation on wheat plants infected with BSMV-γ and BSMV-TaSWI3B. Bar, 150 µm.
